# Treatment of persistent insomnia in patients with benzodiazepine addiction: a case report

**DOI:** 10.1192/j.eurpsy.2025.2329

**Published:** 2025-08-26

**Authors:** A. Castiglioni García-Diego, A. Blanco Barrón, M. T. González Salvador, M. Magariños López, I. Álvarez Correa

**Affiliations:** 1 Psiquiatría; 2 Hospital Universitario Puerta de Hierro Majadahonda, Madrid, Spain

## Abstract

**Introduction:**

The diagnosis of persistent insomnia according to DSM-5 and ICD 11 is based on clinical criteria, specially in relation to dissatisfaction with the quantity/quality of sleep and the discomfort or impairment of social and occupational functioning that it generates. On the other hand, in 2023, Spain has positioned itself as the world leader in the consumption of benzodiazepines.

We present the case of a 62-year-old man admitted to neurosurgery for a cerebrospinal fluid fistula and evaluated by the liaison psichiatry for persistent insomnia. The patient was abusing benzodiazepines to improve his night’s rest. Different drugs are prescribed to improve sleep and avoid abusive consumption of benzodiazepines such as trazodone, quetiapine, clotiapine, gabapentin with little or no improvement of insomnia. Finally, a dual orexin receptor antagonist, daridorexant, is prescribed and its effectiveness is evaluated.

**Objectives:**

The aim of this work is to evaluate the effectiveness of daridorexant in persistent insomnia, as well as to assess the possibility of its use in benzodiazepine addiction for hypnotic purposes.

**Methods:**

The Oviedo Sleep Questionnaire (COS), which is a structured and hetero-applied instrument, was administered to facilitate the diagnosis and monitoring of sleep pre and post treatment.

A literature search was carried out in PubMed and the conclusions found in the literature were compared with the clinical case presented.

Permission is requested from the patient to present this case anonymously.

**Results:**

After 30 days of treatment with daridorexant, the COS score decreased, and improvement was also observed in the anamnesis, without verbalizing complaints that could be interpreted as side effects.

Regarding lorazepam consumption, this could be gradually reduced until its withdrawal.

**Conclusions:**

Daridorexant is a drug that may be effective in resolving persistent insomnia in patients addicted to benzodiazepines for hypnotic purposes.

The review of the available literature suggests that daridorexant is a safe and effective option for the treatment of persistent insomnia. This has been fulfilled in our patient, given that he has not reported side effects, that the analytical parameters have not changed since its introduction and that it has improved in general terms the quality of sleep and functionality during the day. On the other hand, since the hypnotic purpose of lorazepam was replaced by daridorexant, the former could be withdrawn, which opens the door to the possibility that we are dealing with a drug that reduces the consumption of benzodiazepines.

It should be borne in mind that in Spain daridorexant was recently approved in September 2023, with not much experience in practice.

**Disclosure of Interest:**

None Declared

